# Factors impacting producer marketing through community supported agriculture

**DOI:** 10.1371/journal.pone.0219498

**Published:** 2019-07-09

**Authors:** Huan Dong, Benjamin Campbell, Adam N. Rabinowitz

**Affiliations:** 1 School of Public Administration, Sichuan University, Chengdu, Sichuan, China; 2 Department of Agricultural and Applied Economics, University of Georgia, Athens, Georgia, United States of America; 3 Department of Agricultural and Applied Economics, University of Georgia, Tifton, Georgia, United States of America; University of Florida, UNITED STATES

## Abstract

Nowadays, consumers have become increasingly aware of their local food system as a result of concerning about health and nutrition, food safety and sustainability, and local economic development. This transitional shift from global to direct-to-consumer farm operations has increased the demand for locally produced foods. As an alternative, community supported agriculture (CSA), a direct and sustainable food channel, has gained tremendous popularity in the US. Despite the interest garnered by local agriculture and CSA, relatively few studies have empirically tested the determinants of why this marketing phenomenon has grown so rapidly. The purpose of this study is to explore the factors that drive producers to market their products through CSA by using a county-level data set from the US. Results using a Tobit model indicate that specific operator characteristics, such as young and female operators and those engaged in farming as primary occupation, play a strongly positive role in the likelihood of marketing through CSA; farms with small size, rented land, and engagement in growing vegetables, melons, fruits and tree nut crops are more interested in marketing via CSA; households with higher income and females significantly increase the share of farms marketing through CSA; presence of children and seniors and being married are negatively related to the demand for CSA foods. Moreover, counties with higher density of population, establishments-supermarket and other grocery stores, and legislation or active programs that encourage local food consumption tend to encourage more farms marketing through CSA.

## Introduction

Although the industrialization and globalization of food system can feed a larger world population, it also brings food quality, safety, and sustainability issues to the forefront. Over the past decades, many consumers have become increasingly aware of their local food system as a result of concerning about health and nutrition, food safety and sustainability, and local economic development [1]. This transitional shift from global to direct-to-consumer farm operations has increased the demand for locally produced foods [2–3]. As the local food movement has grown, community supported agriculture (CSA) has expanded to provide a direct link between local farmers and consumers. The first CSA farm in the US was established in 1986 [4]. As an alternative, direct, and sustainable food channel, CSA is gaining tremendous popularity in the US. In 2012, the number of farms marketing their products through CSA had expanded to nearly 12,617 [5].

Academics have analyzed CSA in many ways. Numerous studies about CSA have focused on the consumer perspective. Some studies have examined the motivations for consumers joining CSA [6–10], and the traits of consumers who prefer to obtain local foods through CSA-like arrangements [11]. Some studies have empirically analyzed the factors influencing the probability of becoming a CSA member. Studies found that increasing cost of membership and share of children under 18 years of age decrease the probability of joining CSA [4]. Lack of product choice, the amount of produce provided, as well as transportation and inconvenience of pickup place or time are also limiting factors to joining; however, higher education and a preference for organic products are positively linked to CSA membership [12]. Vassalos et al. [13] indicated that the factors affecting current and future CSA participation differ significantly, specifically, none of the demographic characteristics and the information outlets examined affect current CSA participation, word-of-mouth and online sources significantly affect future CSA participation, lifestyle preferences have a significant impact on current and future CSA participation. Some other studies have investigated CSA’s impact on consumers. Perez et al. [14] and Curtis et al. [15] found that CSA member are likely to have a healthier food consumption pattern. Berning [16] indicated that CSA food has a negative association with individual weight outcomes. Several studies also highlight the psychological benefits of joining CSA [8, 17–18].

Some studies have also considered the social and ecological effects of CSA [19–21]. For example, Van der Ploeg et al. [22] concluded that CSA farms play an important role in developing a more sustainable agriculture system and rebuilding viability of rural community. Paul [23] found that CSA farming results in several non-monetary benefits, including community building, ecosystem services, healthy food, and education.

Compared to numerous studies on consumer and community perspectives, there is considerably less research on CSA in farmers’ own words. Some studies have analyzed the challenges faced by CSA operators, such as insufficient farm income [24] and staggered crops, where everything matures at different times and yield is produced throughout the entire growing season instead of in periodic bunches [25]. Some studies have investigated the factors affecting the development of CSA. For example, Meyer [26] found that market rivals, new entrants, CSA members, farm inputs, and substitutes were core competencies that could significantly affect the development of CSA. Connolly and Klaiber [27] concluded that there was a positive relationship between the number of CSA farms and total population, mean households income, household size, and share of children. Others found that CSA farmers had higher gross and net farm incomes than non-CSA farmers [23,28].

Some works that did not directly focus on CSA are still useful to our study. Uematsu and Mishra [29] indicated that direct marketing strategy is a risk management tool rather than a profit-maximizing strategy. Park et al. [30] found that both management and marketing skills significantly affect direct-to-consumer sales.

While some studies have provided insights into producers developing CSA, very few have empirically tested the determinants of why this marketing phenomenon has grown so rapidly in recent decades. Specifically, existing studies that focus on producers have narrow geographic scope, and therefore, are potentially limited in their ability to generalize throughout the US. This study attempts to fill this research gap by analyzing the factors that drive producers to market their products through CSA. Unlike previous studies with smaller samples, this study (1) uses a county-level data set from the US, which can help avoid sample selection bias; and (2) assesses the effect of producer and consumer characteristics on marketing via CSA at the county level. The results are useful for policy makers, producers, and stakeholders interested in CSA (and local food) viability.

### Theoretical discussion of marketing through CSA and hypotheses

By definition, CSA is a direct marketing arrangement where consumers purchase “shares” from a farm, with producers’ planting decisions based on an agreed amount of product (typically fresh food) and a set schedule for providing this to consumers during the growing season [31]. As such, the number of farms marketing their products through CSA can be thought of as representing an equilibrium outcome of producers’ supply of CSA products and consumers’ demand for these products.

### Uncertainty, risk aversion, and small-scale farmers’ decision

Due to the fact that uncontrollable elements, such as weather, prices, policy, and technology play a fundamental role in agricultural production, uncertainty and risk are quintessential features of agricultural production [32]. With the expected utility model [33], the decision of a farm to market its products through CSA can be represented as:

$$E_{CSA}[U)w_{0}+{\tilde{\pi }}_{CSA})]\geq E_{OTHER}[U(w_{0}+{\tilde{\pi }}_{OTHER})]$$ (1)

where *OTHER* ∈ *A*, which represents the set of all other possible marketing channels available to farms, such as farmers’ markets, roadside stands, grocery stores, intermediary marketing channels, etc.; *w*_0_ is the initial wealth of the farm; and ${\tilde{\pi }}_{CSA}$ and ${\tilde{\pi }}_{OTHER}$ are profits gained by marketing products through CSA and other channels, respectively, both of which are given by:

$$\tilde{\pi }=\tilde{p}\times Q(x;\tilde{e})-C(x,p)-C_{f}$$ (2)

where $\tilde{p}$ is output price; $Q(x;\tilde{e})$ represents the production function, which depends on the vector of inputs *x* and a vector of random variables $\tilde{e}$; *C*(*x*, *p*) represents a cost function conditional on the vector of inputs *x*, and inputs prices *p*, and *C*_*f*_ are fixed costs.

As a marketing arrangement for farms, CSA has grown substantially in the US over the past decades. Both the absolute and relative number of farms marketing through CSA are increasing [5, 34]. From the producers’ perspective, two main reasons can explain farms’ interest in CSA marketing arrangement.

First, CSA can help farms remain competitive by hedging risk under uncertainty. With a CSA arrangement, producers and consumers agree on terms of output price and delivery in advance. That means when marketing products through CSA, $\tilde{p}$ becomes a constant *P*_*CSA*_ for farms before the farming season begins. In effect, a rational risk-averse producer, whose expected utility is consistent with Eq (1), will choose to market their products through CSA in order to reduce risk. Therefore, we hypothesize that producers with higher risk aversion are more interested in marketing products through CSA.

Second, marketing products through CSA can afford producers the security of a higher margin that is often not achievable without large-scale operations [4]. Due to smaller quantities of products, most small-scale producers lack bargaining power to enter larger markets. Thus, the potential for guaranteed income before growing season makes selling via CSA a viable and attractive alternative.

Therefore, we hypothesize that certain production factors will affect marketing via a CSA:

1. Farms located in areas with younger operators might be more willing to market products through CSA than areas comprising mostly of older farmers. Younger operators might be more interested in new things. Thus, they are more likely to accept CSA philosophy and try it in practice.
2. Farms located in areas with more female operators might be more interested in marketing products through CSA. Women are more risk-averse than men [35–36]. Therefore, they might tend to market their products through CSA, wherein they can receive a certain amount of money before planting begins.
3. Farms located in areas with more operators whose primary occupation is farming, are more likely to market products through CSA. This is because operators whose primary occupation is farming might have a good relationship with the local community and will be more interested in local food and local development.
4. Areas with more small-scale farms are more willing to market through CSA. As discussed above, small-scale farmers lack assets and policies protection, such as subsidies and agricultural insurances. These translate into lower ability to deal with risk. Thus, they might be more interested in CSA, which can share farming risk with consumers, including poor harvests [37–38].
5. Farms primarily engaged in growing vegetables, melons, fruits and tree nut crops, are more likely to market products through CSA. Monson et al. [39] found that farmers specializing in vegetables, fruits and nursery are more likely to engage in direct marketing.

### Food safety issues, health problems, and consumers’ responses

Food should be a source of nourishment. Unfortunately, there are a one in six chance that Americans suffer from a foodborne illness [40]. Due to a global supply chain, it is extremely difficult to trace where food comes from, and how it is grown and prepared. Therefore, a growing percentage of the US population has begun to question the industrialized food system. As an outcome of induced demand, CSA fills this niche market by directly offering fresh and high-quality products to consumers.

Following the microeconomic theory of Jehle and Reny [41], we assume that there are two food channels: CSA foods, and conventional foods, including foods from supermarkets, grocery stores, farmers’ markets and others. For household *i*, *V*(*P*_*CSA*_, *W*, *X*) and *V*(*P*_*CON*_, *W*, *X*) are the indirect utility functions for consumption of a unit of food from CSA and conventional channels, respectively. Here, *P*_*CSA*_ and *P*_*CON*_ denote the price of buying food from CSA and conventional channels, respectively; *W* represents household income; and *X* is a vector of household characteristics, such as household socio-demographic variables [42]. Moreover, we assume that the indirect utility function is continuous, strictly increasing in *W*, and decreasing in *P*. Theoretically, households will purchase food from CSA farms only if the utility of purchasing food from them is at least as good as that from other food channels, which can be expressed as:

$$V(P_{CSA},W,X)\geq V(P_{CON},W,X)$$ (3)

Therefore, food price, household income, and household socio-demographic characteristics are key factors in explaining consumers’ purchase decision. Further, we assume the consumers to believe that CSA poses fewer health risks than conventional food channels. We hypothesize that:

1. The higher the median household income of an area, the more the number of farms that will market via CSA. Given higher incomes, households prefer to purchase foods they believe are safer and of higher quality [43], while also purchasing more fresh vegetables and fruits [44–45].
2. The higher the education level of an area, the more the demand for CSA foods, which in turn would lead to farmers marketing their products through CSA. Highly educated consumers consider themselves more responsible for their own health [46], and hence might be more interested in CSA, which allows food to be traced directly. Existing research has found CSA participants to be highly educated [15].

### Area characteristics, and market rivals

Production and consumption characteristics of an area are not the only factors impacting the decision to market via CSA; area characteristics may also play a pivotal role. Furthermore, it is imperative to understand how a competitive environment impacts producers’ participation in CSA. Therefore, we hypothesize that:

1. Counties with higher population densities would see an increased number of farms marketing via CSA, given the potential to reach more consumers.
2. Counties with more supermarkets and other grocery stores per capita would see less farmers marketing through CSA, since supermarkets and other grocery stores are main market rivals for CSA [26].

## Data

To estimate the impact of production and consumption characteristics on producers’ CSA participation, we collect data from several sources at the county level. The number of farms marketing their products through CSA, total number of farms, number of farms which the principal operator’s age is under 44 years, number of farms which the principal operator’s age is over 60 years, number of farms with woman operators, number of farms with size of 1–9 acres, number of farms with size of 10–49 acres, number of farms with size of 50–179 acres, number of farms classified for fruit and tree nut farming, number of farms classified for vegetable and melon farming, number of farms with their own land, number of farms with partial ownership of land, number of farms with rented land, number of farms which principal operator’s primary occupation is farming, and harvested acres of vegetables for sale, are collected from the 2007 and 2012 Census of Agriculture.

With respect to the consumption characteristics, it is not always possible to match data with the 2007 and/or 2012 years. Unlike production values, the year-to-year variation in consumption characteristics such as median income or population density are generally small. For such cases, we obtain data for the closest available year. Population density, percentage of females, and percentage of children and seniors in a county are collected from the 2005 and 2010 US Census. County-level education, household size, and percentage of households married (except separated couples) with population 15 years and over are obtained from the 2009 and 2012 American Community Survey 5-year estimates, US Census Bureau. Additionally, median household income per county in 2007 and 2012 is taken from the Small Area Income and Poverty Estimates Program, US Census Bureau.

Lastly, the number of supermarkets and other grocery stores in 2007 and 2012 are identified as NAICS 445110 and collected from the County Business Patterns, US Census Bureau.

Table 1 provides variable definitions and summary statistics used in our empirical analysis. Since variables containing missing data for some counties will reduce the representativeness of the sample and therefore affect conclusions, we discard those observations. Moreover, Hawaii is excluded because of lack of data. After eliminating counties with missing data, 4,587 counties remain in our sample, as shown in Table 1.

**Table 1:** Definition, summary statistics, and expected influence direction of variables used in analysis.

Variables	Total		Sample		Influence direction
	Obs	Mean	Obs	Mean	
Dependent variable					
Share of farms marketed through CSA to all farms	6106	0.006	4587	0.007	
Explanatory variables					
Farmer-side characteristics					
Percentage of farms which principal operator’s age is under 44 years	6131	0.164	4587	0.163	+
Percentage of farms which principal operator’s age is over 60 years	6131	0.456	4587	0.454	-
Percentage of women operators	6128	0.299	4587	0.302	+
Percentage of farms with size of 1–9 acres	6131	0.096	4587	0.103	+
Percentage of farms with size of 10–49 acres	6131	0.262	4587	0.290	+
Percentage of farms with size of 50–179 acres	6131	0.295	4587	0.312	?
Percentage of farms classified for fruit and tree nut farming	6131	0.032	4587	0.037	+
Percentage of farms classified for vegetable and melon farming	6131	0.022	4587	0.026	+
Percentage of farms with their own land	6129	0.676	4587	0.692	?
Percentage of farms with partial ownership of land	6118	0.254	4587	0.243	?
Percentage of farms with rented land	6096	0.073	4587	0.066	?
Percentage of farms which principal operator’s primary occupation is farming	6123	0.471	4587	0.460	+
Harvested acres of vegetables for sale	4646	1888	4587	1908	+
Consumer-side characteristics					
Median household income (dollar)	6136	43613	4587	44271	+
Percentage of female	6132	0.502	4587	0.503	+
Percentage of population that is 18 years and over with college or higher education	6136	0.473	4587	0.475	+
Average household size	6136	2.504	4587	2.524	?
Percentage of population under 14 years	6132	0.191	4587	0.193	+
Percentage of population over 65 years	6132	0.155	4587	0.149	-
Percentage of married households	6136	0.539	4587	0.533	+
County and other characteristics					
Population per square mile	6134	182.599	4587	172.017	+
Number of establishments-supermarkets and other grocery stores per population	6021	0.027	4587	0.023	-

## Econometric specification and empirical analysis

The objective of this study is to examine the determinants that may lead to farms marketing their products through CSA. Our dependent variable is the share of farms in a county marketing their products through CSA, which is measured by:

$$S\_CSA=\frac{NUMBER\;OF\;FARMS\;MARKETED\;PRODUCTS\;THROUGH\;CSA}{NUMBER\;OF\;FARMS}\times 100\%$$ (4)

Given that some counties have no farms marketing via CSA, we utilize a Tobit model to account for the left censoring that occurs in the data [47]. The Tobit model is represented as:

$$S\_CSA_{i}^{*}=\beta_{0}+\beta_{k}\times D_{ik}+\delta_{j}\times F_{ij}+\eta_{m}\times C_{im}+\phi_{n}\times dum\_st_{in}^{*}+\gamma \times dum\_2012_{i}+\mu_{i}$$

S_CSAi={0       ifS_CSAi*≤0S_CSAi*    ifS_CSAi*>0 (5)

where, $S\_CSA_{i}^{*}$ for county *i* (*i* = 1,…, *N*) is the censored dependent variable, which is determined by a 1 × *k* vector of observable consumer-side characteristics *D*_*i*_; a 1 × *j* vector of variables *F*_*i*_ representing farmer-side characteristics within the county; a 1 × *m* vector of market related characteristics and county economic indicators *C*_*i*_; and the disturbance term *μ*_*i*_. Furthermore, we take log of “median household income” and “harvested acres of vegetables for sale” to eliminate data heteroscedasticity. We also use state and year dummy variables, which are represented as $dum\_st_{in}^{*}$ and *dum*_2012_*i*_, respectively, to control the effects caused by unobservable differences across states and over time.

The main estimation results for Eq (5) and average marginal effects of the explanatory variables on the share of farms marketing products through CSA are presented in Tables 2 and 3. Marginal effects of the Tobit model help in understanding how a unit change in the explanatory variables affects the share of farms marketing through CSA.

**Table 2:** Parameter estimates and average marginal effects from the Tobit model.

Variable	Coefficient	P>t	Marginal Effect	P>z
Farmer-side characteristics				
Percentage of farms which principal operator’s age is under 44 years	0.036	0.000	1.132	0.000
Percentage of farms which principal operator’s age is over 60 years	0.007	0.085	0.222	0.085
Percentage of women operators	0.038	0.000	1.147	0.000
Percentage of farms with size of 1–9 acres	0.009	0.010	0.279	0.010
Percentage of farms with size of 10–49 acres	0.005	0.049	0.161	0.049
Percentage of farms with size of 50–179 acres	0.011	0.003	0.339	0.003
Percentage of farms classified for fruit and tree nut farming	0.010	0.002	0.316	0.002
Percentage of farms classified for vegetable and melon farming	0.044	0.000	1.388	0.000
Percentage of farms with their own land	0.015	0.107	0.465	0.107
Percentage of farms with partial ownership of land	0.013	0.184	0.407	0.184
Percentage of farms with rented land	0.039	0.000	1.226	0.000
Percentage of farms which principal operator’s primary occupation is farming	0.007	0.016	0.228	0.016
Log (harvested acres of vegetables for sale)	-0.001	0.647	-0.001	0.647
Consumer-side characteristics				
Log (median household income [dollar])	0.004	0.014	0.132	0.014
Percentage of female	0.022	0.080	0.697	0.080
Percentage of population that is 18 years and over with college or higher education	0.002	0.441	0.082	0.441
Average household size	-0.002	0.140	-0.061	0.140
Percentage of population under 14 years	-0.055	0.000	-1.770	0.000
Percentage of population over 65 years	-0.024	0.013	-0.772	0.013
Percentage of married households	-0.013	0.003	-0.419	0.003
County and other characteristics				
Population per square mile (in 1,000)	4.57e-06	0.000	0.001	0.000
Number of establishments-supermarkets and other grocery stores per population	0.083	0.000	2.635	0.000
Number of obs = 4587				
Log likelihood = 10716.098	Prob > chi2 = 0.0000			
Pseudo R2 = -0.1111	LR chi2(71) = 2142.28			

We use Stata software to estimate the model results; dy/dx is for discrete change of dummy variable from 0 to 1.

**Table 3:** Parameter estimates and average marginal effects of state effects (Vermont as Reference State, Reduced Edition).

Variable	State Name	Coefficient	P>t	Marginal Effect	P>z
dum_st2	Alaska	-0.004	0.545	-0.138	0.567
dum_st18	Maine	0.000	0.940	0.006	0.940
dum_st20	Massachusetts	0.010	0.002	0.220	0.000

Hawaii is excluded from the analysis owing to lack of data.

### Small-scale farms primarily engaged in growing vegetables, melons, fruits and tree nut crops, and headed by younger and women operators whose primary occupation is farming, prefer to market products though CSA

The results show that the likelihood of farms marketing products through CSA increase with an increase in the share of younger and women operators; both are statistically significant at the 0.01 level. Their average marginal effect on the share of farms marketing through CSA is 113.2% and 114.7%, respectively. Another interesting finding is that, a unit increase in the percentage of operators aged over 60 years contributes to a greater probability (22.2%) of CSA marketing. Elder operators lack opportunities to work outside farms. They might be more concerned about relationship with local community. Thus, forming a CSA farm might become their choice. As expected, a unit increase in the percentage of operators whose primary occupation is farming leads to a 22.8% increase in the likelihood of marketing through CSA.

In term of production characteristics, small-scale farms are positively related to the share of farms marketing through CSA. Overall, a unit increase in the percentage of the 1–9 acres group, the 10–49 acres group, and the 50–179 acres group increase the likelihood of marketing through CSA by 27.9%, 16.1%, and 33.9%, respectively. Furthermore, a unit increase in the share of farms growing vegetables and melons, and fruits and tree nut crops result in 138.8% and 31.6% higher probability of marketing through CSA, respectively. An unexpected result is that tenants with farms, which are located on land rented from others, are also more likely to market through CSA. A unit increase in the percentage of tenant farms increases the share of producers marketing through CSA by 122.6%.

The acres of vegetables harvested for sale have no influence on the number of farms marketing through CSA. A probable reason is that consumers’ decisions on whether to purchase CSA foods, and producers’ decisions on marketing products through CSA, are made before growing season.

### The best target market catering to the formation of CSA is highly correlated with high-income households comprised of more females, less seniors, and less children

Regarding the effect of income, median household income has a positive influence on the share of farms marketing products through CSA. However, the average marginal effect is small because food expenditure loses participation with respect to total household expenditure.

As expected, number of females is positively correlated to the number of farms marketing through CSA by inducing more demand for CSA foods. A unit increase in the percentage of females contributes to a 69.7% higher probability of marketing through CSA.

A contradictory outcome is that the share of children and seniors are both negatively associated with the share of farms marketing through CSA, and their marginal effect is 177.0% and 77.2%, respectively. The reason is that households spend a lot of time in taking care of children and seniors. Thus, as a time-constraining factor, households including children and seniors may lack the time and energy to join CSA. Moreover, families with children and seniors tend to spend more on education and healthcare, and thus, may have less budget for food consumption, leading to their preference for grocery stores rather than CSAs.

Furthermore, married households are negatively associated with the share of farms marketing through CSA, which is contradictory to our expectation. Married households have more chores to do, which might be why they have less time joining CSA. On the other hand, education and household size have no significant impact on the likelihood of consumers’ demand for CSA foods.

### Density of population, supermarkets, and other grocery stores are positively related to the share of farms marketing through CSA

Population density plays a positive role in explaining the share of farms marketing through CSA, which makes intuitive sense, since higher population density implies greater food demand. However, the marginal effect of population density on the share of farms marketing through CSA is almost zero. A possible reason is that CSA has existed for nearly 30 years in the US, and its philosophy has changed a lot in practice. Nowadays, CSA foods are demanded not only by urban population but also by those who live in the countryside, which is regarded as an area with lower population density.

Unexpectedly, the result shows that the higher the density of establishments-supermarkets and other grocery stores in a county, the more the share of farms that market through CSA. This result indicates that for consumers, food from establishments-supermarkets or other grocery stores is not completely substitutes with food from CSA. Counties with higher density of establishments-supermarkets and other grocery stores imply a bigger market size, which results in increased demand for all types of foods, including CSA foods.

### Compared to Vermont, Maine and Alaska are not significant; Massachusetts is positively associated with the share of farms marketing through CSA; other states have a negative relationship with the share of farms marketing through CSA

Finally, we focus on state-specific variation by creating state dummy variables. We choose Vermont as the reference value, since Vermont is the national leader in local food movement and ranked 1^st^ in consuming locally produced food according to the 2015 Locavore Index by Strolling of the Heifers.

Unsurprisingly, most states are significantly negatively related to the share of farms marketing through CSA than Vermont (see S1 Table for more details). However, farms in Massachusetts have a 22.0% higher willingness to market products through CSA. Recently, local food movement has rapidly expanded in Massachusetts, and the state has moved to 5^th^ place from 11^th^ in 2014 by Strolling of the Heifers 2015 Locavore Index. This could be the reason Massachusetts is more positively related to the share of farms marketed through CSA compared to Vermont.

Maine has been continuously ranked 2^nd^ in the Locavore Index for many years [48], which means that CSA is well established in Maine as well. Thus, compared to Vermont, Maine is not significantly related to the share of farms marketing through CSA. Owing to missing data, Alaska has only four observations in our dataset, and therefore not statistically significant.

## Conclusions

Although CSA represents only a small share of the US food system, the increased focus on food safety, combined with the rise in popularity of local food systems, has heightened interest in the CSA philosophy. In this analysis, we assess what factors affect the share of farms marketing their products through CSA by evaluating the socio-demographic and economic driving forces from various perspectives, using a county-level data set from the US. The results provide helpful implications for policymakers and producers.

As to the government, based on multifunctionality of agriculture, the agricultural policies are not only aimed at economics but also combined with political, ecological, social, and cultural goals. Since CSA activity has become an important part of sustainable local food system, it is more than just a practice of producing and distributing food; it also plays an important role in developing solidarity and cooperative social relationships, and the social and economic life of a community. Thus, it is important for the government to nurture a better development environment for CSA. Results from our study confirm that operator characteristics, such as young and female operators, and operators with farming as primary occupation play a strongly positive role in the likelihood of marketing through CSA; farms with small size, tenants tenure characteristics, and engaged in growing vegetables, melons, fruits and tree nut crops are more likely to market products through CSA. Therefore, we suggest (1) encouraging younger and female agricultural labor to engage in CSA with preferential policy and project support; (2) conducting training courses such as knowledge and awareness of local food resources, marketing skills, etc. for operators with farming as primary occupation to enhance CSA marketing; (3) providing policy support such as direct or guaranteed loans and farm storage facility loans to foster a better development environment for CSA; and (4) giving technical assistance to farms engaged in CSA.

From the producer’s perspective, a CSA marketing arrangement presents a chance for interested operators to tap into this niche market. However, if CSA farms fail to provide viable livelihoods for farm operators, the worth, survival probability, and expansion of the CSA model will be questioned. From the CSA model, it is obvious that the decisive factor for CSA development is its membership base. Therefore, in order to improve CSA farms’ long-term viability, it is important for farm operators to understand CSA’s target market. Our research found that households with higher income and those comprised mostly of females significantly increase the share of farms marketing through CSA; presence of children and seniors and being married are negatively related to the demand for CSA foods. Such results can help CSA producers develop better farm strategy and business plan for various target groups.

However, we treat farms that market products through CSA as homogenous farms, which may result in omission of important factors. Future research from CSA farm-level is needed. In addition, our model did not capture policy variables in detail, and this should be modeled in future research as well.

## Supporting information

S1 Dataset Data-CSA. (XLS)

S1 File Data availability statement-supporting information. (DOCX)

S1 Table Parameter estimates and average marginal effects of state and year effects. (DOCX)
