# Supplementary material for: Factors impacting producer marketing through community supported agriculture
Source: PLoS One. 2019 Jul 9;14(7):e0219498. doi: 10.1371/journal.pone.0219498 (PMC6615704; doi:10.1371/journal.pone.0219498)
Supplement: S1 File — (DOCX) [file pone.0219498.s002.docx]

Data availability statement

All of the data used in our study are collected by authors from different sources. I confirm that those interested can access the data in the same way the authors did and that the authors did not have any special access privileges.

1. The dependent variable is “the share of farms marketed through CSA to all farms” which is measured by “numbers of farms marketed their products through CSA” dividing “number of farms”. Moreover, we have thirteen explanatory variables in farmer-side, which are percentage of farms which principal operators' age is under 44 years (equal to “numbers of farms which principal operators' age is under 44 years” divide “number of farms”), percentage of farms which principal operators' age is over 60 years (equal to “numbers of farms which principal operators' age is over 60 years” divide “number of farms”), percentage of women operators (equal to “numbers of woman operators” divide “number of operators”), percentage of farms with size of 1-9 acres (equal to “numbers of farms with size of 1-9 acres” divide “number of farms”), percentage of farms with size of 10-49 acres (equal to “numbers of farms with size of 10-49 acres” divide “number of farms”), percentage of farms with size of 50-179 acres (equal to “numbers of farms with size of 50-179 acres” divide “number of farms”), percentage of farms which classification is fruit and tree nut farming (equal to “numbers of farms which classification is fruit and tree nut farming” divide “number of farms”), percentage of farms which classification is vegetable and melon farming (equal to “numbers of farms which classification is vegetable and melon farming” divide “number of farms”), percentage of farms who own their land (equal to “numbers of farms who own their land” divide “number of farms”), percentage of farms with partial ownership of land (equal to “numbers of farms with partial ownership of land” divide “number of farms”), percentage of farms who rent their land (equal to “numbers of farms who rent their land” divide “number of farms”), percentage of farms which principal operators' primary occupation is farming (equal to “numbers of farms which principal operators' primary occupation is farming” divide “number of farms”), harvested Acres of vegetables for sale.

The numbers of farms marketed their products through CSA, the number of farms, number of farms which principal operators' age is under 44 years, numbers of farms which principal operators' age is over 60 years, numbers of woman operators, number of operators, numbers of farms with size of 1-9 acres, numbers of farms with size of 10-49 acres, numbers of farms with size of 50-179 acres, numbers of farms which classification is fruit and tree nut farming, numbers of farms which classification is vegetable and melon farming, numbers of farms who own their land, numbers of farms with partial ownership of land, numbers of farms who rent their land, numbers of farms which principal operators' primary occupation is farming, harvested Acres of vegetables for sale are collected from the 2007 and 2012 Census of Agriculture. The relevant link is [*https://www.nass.usda.gov/Publications/AgCensus/2012/Full_Report/Volume_1,_Chapter_2_County_Level/*](https://www.nass.usda.gov/Publications/AgCensus/2012/Full_Report/Volume_1,_Chapter_2_County_Level/) and [*https://www.nass.usda.gov/Publications/AgCensus/2007/*](https://www.nass.usda.gov/Publications/AgCensus/2007/) . You can collect the data by downloading the “desktop data query tool 2.0” and “desktop data query tool 1.02”.

2. We have seven explanatory variables in consumer-side, which are median household income, percentage of female (equal to “population estimate-female” divide “population estimate-both sexes”), percentage of population 18 years and over with college or higher (equal to “population 18 years and over with college or higher” divide “population estimate-both sexes”), average household size, percentage of population under 14 years (equal to “population estimate-under 14 years” divide “population estimate-both sexes”), percentage of population 65 years over (equal to “population estimate-65 years over” divide “population estimate-both sexes”), percentage of married households. With respect to the consumer characteristics, it is not always possible to match data with the 2007 and/or 2012 years. Since unlike production values, consumer demographics such as median income or population density generally vary very little from year to year. In these cases we obtain the closest year available.

Median household income per county in 2007 and 2012 are taken from the Small Area Income and Poverty Estimates Program, U.S. Census Bureau. The relevant link is [*https://www.commerce.gov/taxonomy/term/4*](https://www.commerce.gov/taxonomy/term/4). Data Source: Table Poverty and Median Household Income Estimates - Counties, States, and National, U.S. Census Bureau, Small Area Income and Poverty Estimates (SAIPE) Program.

Population estimate of female, population estimate in both sexes, population estimate under 14 years, population estimate over 65 years are collected from U.S. Census Bureau, Population Division. The relevant link is [*https://www.commerce.gov/taxonomy/term/4*](https://www.commerce.gov/taxonomy/term/4). Specifically, the data source are: Table B-3. Counties -- Population by Age, Race, Hispanic Origin, and Sex 2005”; DP-1 Profile of General Population and Housing Characteristics: 2010.

The data of population 18 years and over with college or higher, household size, percentage of now married (except separated) with population 15 years and over are obtained from the 2009 and 2012 American Community Survey (ACS) 5-year estimates by U.S. Census Bureau. The relevant link is [*https://library.columbia.edu/locations/dssc/data/acs.html*](https://library.columbia.edu/locations/dssc/data/acs.html). Specifically, the data of population 18 years and over with college or higher is collected from “Table S1501-Educational Attainment/”. The data of average household size is collected from “Table S1101-Households and Families”. The data of percentage of now married (except separated)-Population 15 years and over is collected from “Table S1201-Marital Status”.

3. Population per square mile is equal to population divide land area. Specifically, population per square mile was calculated on the basis of land area data from the 2000 census. The data of population is collected from “census population 2010 and 2005”.

4. Lastly, the number of supermarkets and other grocery stores in 2007 and 2012 are identified as the North American Industry Classification System (NAICS) 445110 and collected from the County Business Patterns, U.S. Census Bureau. *Download files can be found at County Business Patterns Website.*
