# Supplementary material for: Factors impacting producer marketing through community supported agriculture
Source: PLoS One. 2019 Jul 9;14(7):e0219498. doi: 10.1371/journal.pone.0219498 (PMC6615704; doi:10.1371/journal.pone.0219498)
Supplement: S1 Table — (DOCX) [file pone.0219498.s003.docx]

**S1 Table. Parameter Estimates and Average Marginal Effects of State and Year Effects (Vermont as Reference State)**

| **Variable** | **State Name** | **Coefficient** | **P>t** | **Marginal Effect** | **P>z** |
| --- | --- | --- | --- | --- | --- |
| dum_st1 | Alabama | -0.020 | 0.000 | -0.625 | 0.000 |
| dum_st2 | Alaska | -0.004 | 0.545 | -0.138 | 0.567 |
| dum_st3 | Arizona | -0.026 | 0.000 | -0.687 | 0.000 |
| dum_st4 | Arkansas | -0.024 | 0.000 | -0.674 | 0.000 |
| dum_st5 | California | -0.020 | 0.000 | -0.622 | 0.000 |
| dum_st6 | Colorado | -0.020 | 0.000 | -0.620 | 0.000 |
| dum_st7 | Connecticut | -0.010 | 0.008 | -0.329 | 0.008 |
| dum_st8 | Delaware | -0.019 | 0.000 | -0.603 | 0.000 |
| dum_st9 | Florida | -0.026 | 0.000 | -0.691 | 0.000 |
| dum_st10 | Georgia | -0.020 | 0.000 | -0.613 | 0.000 |
| dum_st11 | Idaho | -0.019 | 0.000 | -0.604 | 0.000 |
| dum_st12 | Illinois | -0.021 | 0.000 | -0.644 | 0.000 |
| dum_st13 | Indiana | -0.021 | 0.000 | -0.636 | 0.000 |
| dum_st14 | Iowa | -0.020 | 0.000 | -0.621 | 0.000 |
| dum_st15 | Kansas | -0.023 | 0.000 | -0.657 | 0.000 |
| dum_st16 | Kentucky | -0.020 | 0.000 | -0.616 | 0.000 |
| dum_st17 | Louisiana | -0.024 | 0.000 | -0.677 | 0.000 |
| dum_st18 | Maine | 0.000 | 0.940 | 0.006 | 0.940 |
| dum_st19 | Maryland | -0.020 | 0.000 | -0.611 | 0.000 |
| dum_st20 | Massachusetts | 0.010 | 0.002 | 0.220 | 0.000 |
| dum_st21 | Michigan | -0.018 | 0.000 | -0.593 | 0.000 |
| dum_st22 | Minnesota | -0.020 | 0.000 | -0.625 | 0.000 |
| dum_st23 | Mississippi | -0.022 | 0.000 | -0.660 | 0.000 |
| dum_st24 | Missouri | -0.021 | 0.000 | -0.641 | 0.000 |
| dum_st25 | Montana | -0.020 | 0.000 | -0.626 | 0.000 |
| dum_st26 | Nebraska | -0.023 | 0.000 | -0.667 | 0.000 |
| dum_st27 | Nevada | -0.017 | 0.000 | -0.549 | 0.000 |
| dum_st28 | New Hampshire | -0.007 | 0.040 | -0.236 | 0.049 |
| dum_st29 | New Jersey | -0.030 | 0.000 | -0.707 | 0.000 |
| dum_st30 | New Mexico | -0.019 | 0.000 | -0.608 | 0.000 |
| dum_st31 | New York | -0.017 | 0.000 | -0.571 | 0.000 |
| dum_st32 | North Carolina | -0.017 | 0.000 | -0.567 | 0.000 |
| dum_st33 | North Dakota | -0.028 | 0.000 | -0.696 | 0.000 |
| dum_st34 | Ohio | -0.020 | 0.000 | -0.628 | 0.000 |
| dum_st35 | Oklahoma | -0.022 | 0.000 | -0.660 | 0.000 |
| dum_st36 | Oregon | -0.019 | 0.000 | -0.610 | 0.000 |
| dum_st37 | Pennsylvania | -0.021 | 0.000 | -0.636 | 0.000 |
| dum_st38 | Rhode Island | -0.014 | 0.000 | -0.494 | 0.000 |
| dum_st39 | South Carolina | -0.018 | 0.000 | -0.577 | 0.000 |
| dum_st40 | South Dakota | -0.023 | 0.000 | -0.657 | 0.000 |
| dum_st41 | Tennessee | -0.021 | 0.000 | -0.647 | 0.000 |
| dum_st42 | Texas | -0.021 | 0.000 | -0.654 | 0.000 |
| dum_st43 | Utah | -0.013 | 0.000 | -0.459 | 0.000 |
| dum_st45 | Virginia | -0.019 | 0.000 | -0.600 | 0.000 |
| dum_st46 | Washington | -0.019 | 0.000 | -0.594 | 0.000 |
| dum_st47 | West Virginia | -0.020 | 0.000 | -0.629 | 0.000 |
| dum_st48 | Wisconsin | -0.020 | 0.000 | -0.628 | 0.000 |
| dum_st49 | Wyoming | -0.021 | 0.000 | -0.632 | 0.000 |
| dum_2012 |  | 0.001 | 0.059 | 0.026 | 0.059 |
